# 

**Published:** 1894-02-17

**Authors:** 


CONTENTS.

**The Health of the Navy** - 341
**Around the Hospitals** - 342
**Diuretin and Diuresis—The Theoretical Side.** By Sir BENJAMIN WARD RICHARDSON, M.D., F.R.S. - 343
**Studies in Applied Sociology.** III.—The Corpus Sanum - 344
**Current Medical Topics.**—The Treatment of Disease by Animal Extracts - 345
**Practical Aspects of Medical Science** - 346
**Annotations.**—A Reverend Nottingham Surgeon—The Drink Cure at Dundee—Are Anarchists Lunatics? - 347
**Notes and News** - 348
**Scraps and Gleanings** - 360
**The Book World of Medicine and Science** - 361
**Medical Progress and Hospital Clinics**—The Electrolytic Treatment of Hirsuties and of Trichiasis - 349
Modern Methods of Treatment of Simple Fractures - 351
MEDICAL PROGRESS—Nervous Diseases - 352
PROGRESS IN SURGERY - 354
NEW DRUGS AND PREPARATIONS - 355
NEW APPLIANCES AND THINGS MEDICAL - 356
**The Institutional Workshop—**
WITHIN THE HOSPITALS—West Ham, Stratford, and South Essex Hospital - 357
THE STORY OF THE INSANE FROM YEAR TO YEAR - 358
PRACTICAL DEPARTMENTS - 359
HOSPITAL FESTIVALS AND MEETINGS—CONSTRUCTION NOTES - 359

EXTRA SUPPLEMENT.—THE NURSING MIRROR.

**News from the Nursing World.**—Princess Beatrice's Daughter—Additions and Improvements—Uncommonly Hopeful!—A Successful Society—An Excellent Result—A Friend to Nurses—Courses of Lectures—Intelligent Observation—Conflicting Claims—Where are the Nurses?—The American Amateur—Co-operation for Male Nurses - cxci
**On Nursing the Nervous and Insane.** VI.—Idiocy and Imbecility - cxciii
**Notes and Queries** - cxciii
**District Nursing.**—VII. Maternity Work - cxciv
**Nurses' Home, Prince Alfred Hospital, Sydney, New South Wales** - cxcv
**Women's Work.—Artists—Minor Appointments** - cxcvi
**Old-Time Nurses.** I. A Plea for the Middle Aged - cxcvii
**The Nurses' Confessional—Domineering Nurses** - cxcviii
**The Muses' Looking Glass.**—Making Acquaintance with Microbes - cxcix
**Everybody's Opinion—For Reading to the Sick** - cc

